# Neutrophil-to-lymphocyte ratio, white blood cell, and C-reactive protein predicts poor outcome and increased mortality in intracerebral hemorrhage patients: a meta-analysis

**DOI:** 10.3389/fneur.2023.1288377

**Published:** 2024-01-15

**Authors:** Peixin Guo, Wei Zou

**Affiliations:** ^1^Integrated Traditional Chinese and Western Medicine, Heilongjiang University of Traditional Chinese Medicine, Harbin, China; ^2^Third Ward of Acupuncture Department, First Affiliated Hospital, Heilongjiang University of Chinese Medicine, Harbin, China

**Keywords:** intracerebral hemorrhage, neutrophil to lymphocyte ratio, white blood cell, C-reactive protein, prognosis

## Abstract

**Objective:**

Inflammation participates in the pathology and progression of secondary brain injury after intracerebral hemorrhage (ICH). This meta-analysis intended to explore the prognostic role of inflammatory indexes, including neutrophil-to-lymphocyte ratio (NLR), platelet-to-lymphocyte ratio (PLR), white blood cell (WBC), and C-reactive protein (CRP) in ICH patients.

**Methods:**

Embase, PubMed, Web of Science, and Cochrane Library were searched until June 2023. Two outcomes, including poor outcome and mortality were extracted and measured. Odds ratio (OR) and 95% confidence interval (CI) were presented for outcome assessment.

**Results:**

Forty-six studies with 25,928 patients were included in this meta-analysis. The high level of NLR [OR (95% CI): 1.20 (1.13–1.27), *p* < 0.001], WBC [OR (95% CI): 1.11 (1.02–1.21), *p* = 0.013], and CRP [OR (95% CI): 1.29 (1.08–1.54), *p* = 0.005] were related to poor outcome in ICH patients. Additionally, the high level of NLR [OR (95% CI): 1.06 (1.02–1.10), *p* = 0.001], WBC [OR (95% CI): 1.39 (1.16–1.66), *p* < 0.001], and CRP [OR (95% CI): 1.02 (1.01–1.04), *p* = 0.009] were correlated with increased mortality in ICH patients. Nevertheless, PLR was not associated with poor outcome [OR (95% CI): 1.00 (0.99–1.01), *p* = 0.749] or mortality [OR (95% CI): 1.00 (0.99–1.01), *p* = 0.750] in ICH patients. The total score of risk of bias assessed by Newcastle-Ottawa Scale criteria ranged from 7–9, which indicated the low risk of bias in the included studies. Publication bias was low, and stability assessed by sensitivity analysis was good.

**Conclusion:**

This meta-analysis summarizes that the high level of NLR, WBC, and CRP estimates poor outcome and higher mortality in ICH patients.

## 1 Introduction

Intracerebral hemorrhage (ICH) is the second most common type of stroke, which accounts for approximately 27.9% of all incident strokes (1, 2). The global incidence of ICH ranges from 27 to 30 per 100,000 person-years, and the predominant risk factors for ICH include hypertension, coagulopathy, alcohol abuse, diabetes mellitus, smoking, etc. (3–5). Currently, several treatment strategies have been developed to treat ICH patients, such as surgery, blood pressure control, and hemostatic therapy; these therapeutic strategies have made non-negligible progress in treating ICH patients (6–8). Unfortunately, there is no single treatment that effectively improves the prognosis of these patients (9). It is estimated that the mortality after ICH is around 30 to 40% within the first month, and it is approximately 50% within 1 year (10–13). In addition, most patients experience functional decline, and only 12 to 39% of ICH patients achieve long-term functional independence (10, 14, 15). Therefore, identifying potential prognostic factors may be meaningful to enhance the management of ICH patients.

Neutrophils, lymphocytes, platelets, and CRP play a fundamental role in regulating inflammation after ICH, which would further aggravate brain injury and lead to a poor prognosis (16–23). For instance, neutrophils are the first leukocyte subtype to infiltrate into the brain after ICH, which facilitates brain injury by producing reactive oxygen species and releasing proinflammatory cytokines (19). Regarding lymphocytes, ICH would increase catecholamine and steroids to induce lymphocytopenia, which contributes to immunosuppression and aggravates brain injury (20). Besides, platelets are activated after ICH, then they could interact with macrophages to facilitate the production of proinflammatory cytokines, aggravating the brain injury (21). Moreover, C-reactive protein (CPR) could facilitate the production of inflammatory cytokines and induce blood–brain barrier disruption to aggravate inflammation and brain injury (22, 23). Considering their close engagement in ICH, it might be meaningful to explore the prognostic values of relevant inflammatory indicators, including neutrophil-to-lymphocyte ratio (NLR), platelet-to-lymphocyte ratio (PLR), white blood cell (WBC), and CPR in ICH patients (24–27).

One previous study indicates that the high level of NLR is correlated with poor outcome in ICH patients (27). Meanwhile, another study elucidates that the high level of PLR predicts poor outcome, but it cannot estimate mortality in ICH patients (26). Regarding the high level of WBC, it could forecast increased mortality and poor outcome in ICH patients (25). Furthermore, the high level of CRP is associated with elevated mortality and poor outcomes in ICH patients (24). Notably, one recently published meta-analysis has revealed the prognostic role of NLR for ICH patients, which discovers that NLR is correlated with a poor outcome and mortality in ICH patients (28). However, the most recent articles included in this previous meta-analysis are published in 2021, and some updated relevant studies should be considered (28). On the other hand, the previous meta-analysis mainly focuses on the prognostic effect of NLR for ICH patients (28), and whether other inflammatory markers have the same prognostic implication should be further investigated. Accordingly, this meta-analysis enrolled some up-to-date studies and aimed to explore the predictive role of NLR, PLR, WBC, and CRP for poor outcome and mortality in ICH patients.

## 2 Methods

### 2.1 Data sources and searches

Embase, PubMed, Web of Science, and Cochrane Library were searched until June 2023 using the following keywords or a term of their combination: ‘neutrophil-to-lymphocyte ratio’, ‘neutrophil lymphocyte ratio’, ‘neutrophil to lymphocyte ratio’, ‘neutrophil/lymphocyte’, ‘neutrophil-lymphocyte’, ‘NLR’, ‘platelet-to-lymphocyte ratio’, ‘platelet lymphocyte ratio’, ‘platelet to lymphocyte ratio’, ‘platelet/lymphocyte’, ‘platelet-lymphocyte’, ‘PLR’, ‘C-reactive protein’, ‘CRP’, ‘inflammation’, ‘WBC’, ‘WCC’, ‘white cell count’, ‘white blood cell’, ‘leukocyte’, ‘ICH’, ‘intracerebral hemorrhage’, ‘intracranial hemorrhage’, ‘cerebral hemorrhage’, and ‘brain hemorrhage’. The PICOS (Participants, Intervention/exposure, Comparison, Outcomes, Study design) criteria were used to structure this meta-analysis (29). (i) Patients (P): patients diagnosed with ICH. (ii) Intervention (I): patients with a high level of NLR, PLR, WBC, and CRP. (iii) Control (C): patients with a low level of NLR, PLR, WBC, and CRP. (iv) Outcomes (O): poor outcome and mortality. (v) Study design: observational studies.

### 2.2 Outcomes

In this meta-analysis, two outcomes were measured including poor outcome and mortality. Specifically, poor outcome was defined as recording a modified Rankin scale (mRS) score > 2 and/or a Glasgow outcome scale (GOS) score < 4 during the follow-up; and mortality was defined as any cause-death during the follow-up.

### 2.3 Identification criteria

Studies met the following criteria were included: (i) patients diagnosed with ICH; (ii) patients aged more than 18 years; (iii) studies reported inflammation indexes, which contained NLR, PLR, WBC, and CPR (at least one involved); (iv) studies reported multivariate analysis results for outcomes, which contained odds ratio (OR) and corresponding 95% confidence interval (CI). The exclusion criteria were: (i) meeting abstract, letter to the editor, case report, or animal study; (ii) with the non-accessible full-text article; (iii) studies were not English language published. Studies were identified by two independent reviewers (Guo and Zou) in accordance with the above criteria. Disagreements were solved by a consensus of the above two reviewers.

### 2.4 Data extraction and quality assessment

Year, first author, country, study design, number and sex ratio, age, sample time, follow-up period, inflammation indexes, and outcomes were extracted from included studies. The quality of included studies was assessed using the Newcastle-Ottawa scale (NOS) (upper limit, 9; ≥6, high-quality) (30). Besides, data extraction and quality assessment were completed by two independent reviewers (Guo and Zou).

### 2.5 Statistics

The OR with 95% CI related to inflammation indexes and outcomes was calculated. In a meta-analysis, the differences in study design, population, and measurements across different studies were referred to as heterogeneity. For heterogeneity assessment, *I*^2^ test and Q test were used. *I*^2^ represented the ratio of studies heterogeneity to total variation; while Q followed a *χ*^2^ distribution with k-1 degrees of freedom. The range of *I*^2^ values varied from 0 to 100%, with higher values indicating greater heterogeneity. *I*^2^ > 50.0% and *p* < 0.05 ( $Q>\chi_{0.05,k-1}^{2}$ ) were considered as heterogeneity existed, and the random-effect model was used; otherwise, the fixed-effect model was used. Publication bias was shown via Deeks’ funnel plots (Begg’s test). The funnel plots determined the presence or absence of publication bias in meta-analysis based on the degree of asymmetry of the graph. The *p* value of Begg’s test less than 0.05 indicated publication bias existed. If there was a risk of bias, trim and fill analysis was used for further investigation. Sensitivity analysis was used to assess the robustness and reliability of the results by using the leave-one-out approach. If the results of model remain unchanged after sensitivity analysis, the results were reliable. Stata v.14.0 (Stata Corp, USA) was used, and *p* < 0.05 indicated significance.

## 3 Results

### 3.1 Study screening procedure

**Figure 1:**
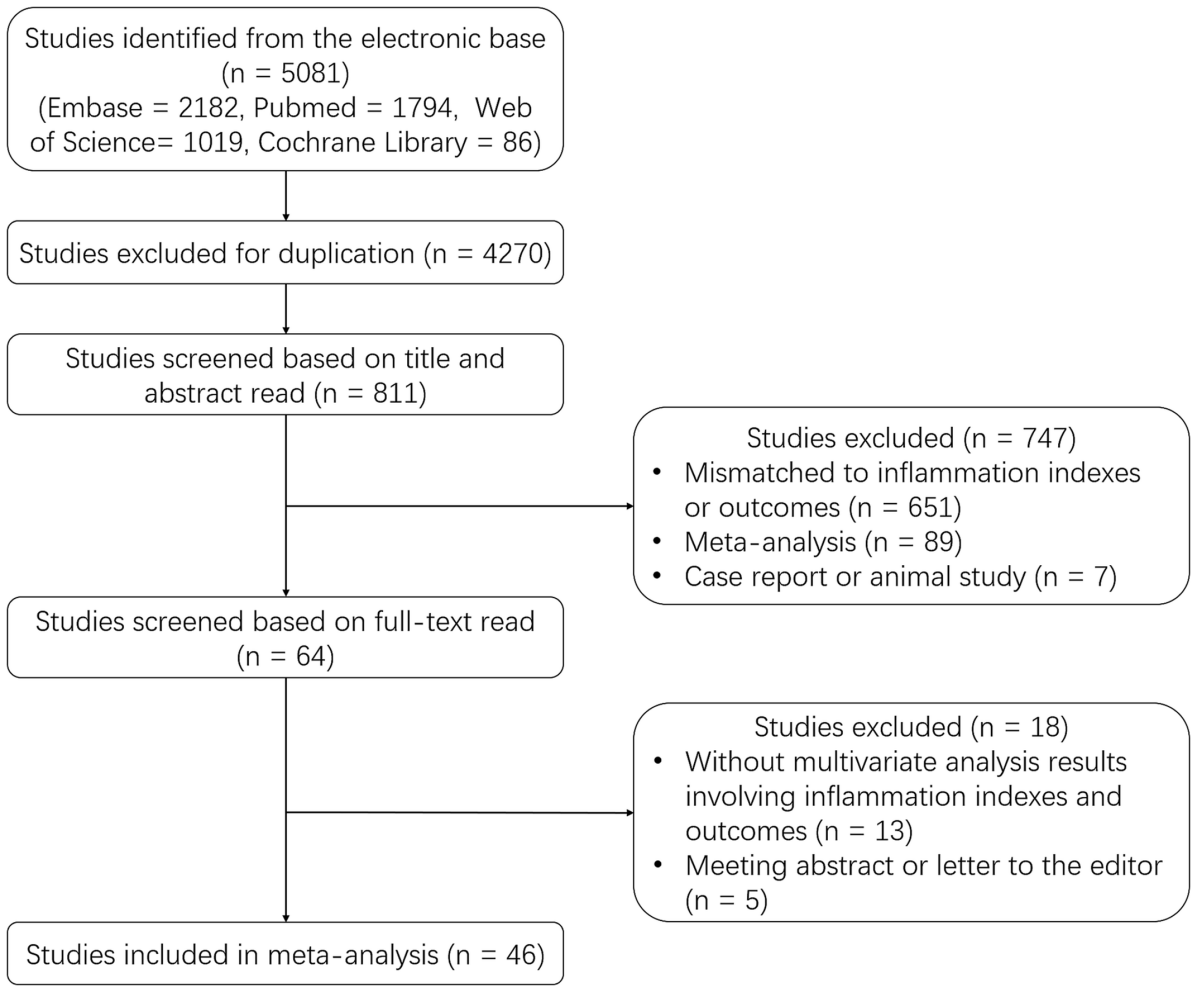
Study flow.

A total of 5,081 studies were identified from the electronic base, including 2,182 studies from Embase, 1794 studies from PubMed, 1,019 studies from Web of Science, and 86 studies from Cochrane Library. Then 4,270 duplicate studies were excluded, and the rest 811 studies were screened based on the title and abstract read. After that, 747 studies were further excluded, including 651 studies that were mismatched to inflammation indexes or outcomes, 89 meta-analyses, and 7 case reports or animal studies. Subsequently, 64 studies were screened based on full-text read, and 18 studies were excluded, including 13 studies without multivariate analysis results and 5 meeting abstracts or letters to the editor. Ultimately, 46 studies were included in this meta-analysis (Figure 1).

### 3.2 Features of included studies

The included studies were published from 2009 to 2023, which contained a total of 25,928 patients (24–27, 31–72). Twenty-five studies were conducted in China, 4 studies were conducted in Italy, 4 studies were conducted in the United States of America (USA), 3 studies were conducted in Germany, 2 studies were conducted in Korea, and the other studies were conducted in Bulgaria, Spain, Finland, Portugal, Romania, Turkey, Tunisia, and India, respectively. The follow-up duration ranged from 30 days to 1 year. The detailed information of the included studies is shown in Table 1.

**Table 1:** Included studies.

Study	Country	Design	Number (M/F)	Age	Sample time	Follow-up	Inflammation indexes	Outcomes
Diedler et al. (31)	Germany	Retro	103 (78/25)	66.6 ± 11.5	Admission	1 year	CRP	1-year poor outcome (mRS 3–6)
Alexandrova and Danovska (32)	Bulgaria	Pro	46 (23/23)	63.0 ± 12.0	Admission	NM	CRP	First-week mortality
Di Napoli et al. (33)	Italy	Pro	210 (122/88)	67.3 ± 11.5	Admission	30 days	WBC; CRP	30-day mortality
Rodríguez-Yáñez et al. (34)	Spain	Retro	141 (66/75)	75.9 ± 12.3	Admission	90 days	WBC; CRP	30-day poor outcome (mRS >2)
Löppönen et al. (35)	Finland	Pro	436 (235/201)	69.0 ± 12.0	In the emergency department or on next morning	90 days	CRP	90-day poor outcome (GOS 1–4)
Adeoye et al. (36)	USA	Retro	186 (94/92)	67.3 ± 14.8	Admission	30 days	WBC	30-day mortality
Walsh et al. (37)	USA	Pro	240 (148/92)	62.8 ± 14.0	Admission	30 days	WBC	30-day mortality
Yu et al. (38)	Korea	Retro	2,630 (1,639/991)	63.7 ± 12.8	Admission	90 days	WBC	90-day poor outcome (mRS 3–6); 90-day mortality
Lattanzi et al. (39)	Italy	Retro	177 (63/114)	67.1 ± 12.5	Admission	90 days	NLR; WBC	90-day poor outcome (mRS ≥3)
Wang et al. (40)	China	Retro	224 (141/83)	68.0 ± 13.8	Admission	30 days	NLR	30-day mortality
Yan et al. (24)	China	Pro	112 (66/46)	63.2 ± 9.6	Admission	180 days	CRP	180-day poor outcome (mRS >2); 180-day mortality
Giede Jeppe et al. (41)	Germany	Retro	855 (457/398)	72.5 (61.0–80.0) for NLR ≥4.7; 71.0 (62.0–78.0) for NLR <4.7	Admission	90 days	NLR	30-day poor outcome (mRS 4–6); 30-day mortality
Tao et al. (25)	China	Retro	336 (216/120)	58.5 ± 13.0	Admission	90 days	NLR; WBC	90-day poor outcome (mRS ≥3); 90-day mortality
Sun et al. (42)	China	Retro	352 (234/118)	64.2 ± 13.8	Admission	90 days	NLR	90-day poor outcome (mRS ≥3); 90-day mortality
Bolayir et al. (43)	Turkey	Retro	296 (138/158)	76.3 ± 11.4	Admission	60 days	CRP	60-day mortality
Elhechmi et al. (44)	Tunisia	Retro	91 (56/35)	64.4 (61.5–67.2)	Admission	30 days	CRP	30-day mortality
Lattanzi et al. (45)	Italy	Retro	208 (132/76)	66.7 ± 12.4	Admission	30 days	NLR; WBC	90-day poor outcome (mRS ≥3)
Fan et al. (46)	China	Retro	225 (176/49)	53.2 ± 10.7	Admission	90 days	NLR; PLR; WBC	90-day poor outcome (GOS <3)
Wang et al. (47)	China	Retro	181 (112/69)	65.8 ± 14.3	Admission	30 days	NLR; CRP	30-day mortality
Qi et al. (48)	China	Retro	558 (368/190)	57.6 (28.0–79.0)	Admission	90 days	NLR; WBC	90-day mortality
Zhang et al. (49)	China	Retro	104 (80/24)	50.4 ± 9.9	Admission	90 days	NLR	90-day poor outcome (GOS ≤3)
Guo et al. (50)	China	Retro	171 (94/77)	46.1 ± 17.3	Admission	90 days	NRL	90-day poor outcome (GOS ≤3)
Qin et al. (51)	China	Retro	213 (157/56)	50.0 (46.0–55.0)	Admission	90 days	NLR	90-day poor outcome (mRS 3–6)
Wang et al. (52)	China	Retro	275 (207/68)	69.0 (53.0–79.0) for the survived; 71.0 (52.0–82.0) for the died	Admission	30 days	NLR	30-day mortality
Zhang et al. (53)	China	Retro	175 (124/51)	60.1 ± 13.0	Admission	30 days	NLR; WBC	30-day poor outcome (GOS <3)
Zhang et al. (54)	China	Retro	481 (350/131)	61.1 ± 12.1	Admission	180 days	NLR; WBC	180-day poor outcome (GOS <3); 180-day mortality
Zhang et al. (55)	China	Retro	107 (72/35)	54.7 ± 12.0	Admission	30 days	NLR; WBC	30-day poor outcome (GOS <3)
Chen et al. (56)	China	Retro	380 (255/125)	58.7 ± 11.4	Admission	30 days	NLR	30-day mortality
Sagar et al. (57)	India	Pro	250 (162/88)	54.9 ± 12.8	Admission	90 days	CRP	90-day poor outcome (mRS 4–6)
Menon et al. (58)	Italy	Retro	851 (604/247)	58.1 ± 12.9	Admission	30 days	NLR	30-day poor outcome (mRS 4–6)
Gusdon et al. (59)	USA	Pro	500 (278/222)	59.0 (51.0–67.0)	Admission	180 days	NLR; WBC	180-day poor outcome (mRS 4–6)
Fonseca et al. (26)	Portugal	Retro	135 (69/66)	73.0 (64.0–80.0)	Admission	90 days	NLR; PLR; CRP	90-day poor outcome (mRS ≥3); 30-day mortality
Mackey et al. (60)	USA	Retro	593 (322/271)	NM	Within 24 h of disease onset	30 days	NLR; WBC	30-day mortality
Li et al. (61)	China	Retro	403 (276/127)	58.6 ± 13.3	Admission	90 days	NLR	90-day poor outcome (mRS ≥3); 30-day mortality
Radu et al. (62)	Romania	Retro	201 (111/90)	70.0 (61.0–79.0)	Admission	30 days	NLR; CRP	In-hospital mortality
Yang et al. (63)	China	Retro	431 (299/132)	58.8 ± 12.9	Admission	90 days	NLR	90-day poor outcome (mRS ≥3); 30-day mortality
Bender et al. (64)	Germany	Retro	329 (177/152)	67.4 ± 13.6	Admission	NM	CRP	In-hospital mortality
Luo et al. (65)	China	Retro	329 (210/119)	61.0 ± 12.6	Admission	90 days	NLR	90-day poor outcome (mRS 4–6)
Zhao et al. (27)	China	Retro	128 (88/40)	60.0 (50.0–67.0)	Within 48 h after surgery	90 days	NLR	30-day poor outcomes (mRS 4–6)
Du et al. (66)	China	Pro	594 (423/171)	56.0 (49.0–64.0)	Admission	90 days	NLR	90-day poor outcome (mRS 3–6); 90-day mortality
Wang et al. (67)	China	Retro	9,589 (6,086/3503)	62.7 ± 13.3	Admission	NM	CRP	In-hospital mortality
Chu et al. (68)	China	Retro	455 (332/123)	62.3 ± 13.4	Admission	90 days	WBC	90-day poor outcome (mRS 4–6); 30-day mortality
Zhang et al. (69)	China	Retro	901 (631/270)	58.7 ± 14.3	Admission	90 days	NLR	90-day mortality
Zhang et al. (70)	China	Retro	101 (69/32)	59.0 (53.5–66.0)	Within 48 h after surgery	30 days	NLR; PLR	30-day poor outcomes (mRS ≥3)
Shi et al. (71)	China	Retro	105 (69/36)	52.6 ± 13.9	Admission	30 days	NLR; WBC; CRP	30-day mortality
Kim et al. (72)	Korea	Pro	520 (312/208)	64.2 ± 15.7	Admission	90 days	NLR; PLR	90-day poor outcome (mRS 3–6); 30-day mortality

M/F, male/female; Retro, Retrospective; CRP, C-reactive protein; mRS, modified Rankin scale; Pro, prospective; NM. Not mentioned; WBC, white blood cell count; GOS, Glasgow outcomes scale; NLR, neutrophil-to-lymphocyte ratio; PLR, platelet-to-lymphocyte ratio. The age was described as mean ± SD or median (interquartile range).

### 3.3 NLR for predicting poor outcome and mortality

**Figure 2:**
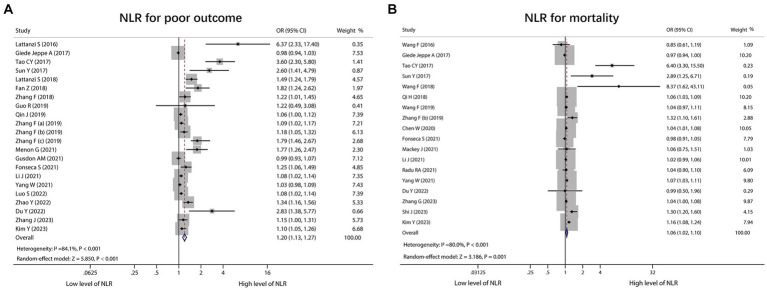
Forest plot of NLR for predicting poor outcome and mortality in ICH patients. Correlation of NLR with poor outcome **(A)** and mortality **(B)** in ICH patients.

A total of 22 studies reported NLR for predicting poor outcome, and heterogeneity existed among these studies (*I*^2^ = 84.1%, *p* < 0.001). The pooled analysis disclosed that the high level of NLR was related to poor outcome in ICH patients [OR (95% CI): 1.20 (1.13–1.27), *p* < 0.001] (Figure 2A). In terms of mortality, 18 studies reported the association of NLR with mortality, and heterogeneity existed among these studies (*I*^2^ = 80.0%, *p* < 0.001). The pooled analysis suggested that the high level of NLR was linked with increased mortality in ICH patients [OR (95% CI): 1.06 (1.02–1.10), *p* = 0.001] (Figure 2B). Two studies clearly indicated that they excluded aneurysmal cerebral hemorrhage patients. Thus, a subgroup analysis was carried out based on these 2 studies. It was found that no heterogeneity existed between these 2 studies (*I*^2^ = 67.6%, *p* = 0.079). The pooled analysis discovered that the high level of NLR showed a trend to correlate with increased mortality in ICH patients, but it did not achieve statistical significance [OR (95% CI): 1.11 (0.99, 1.23), *p* = 0.065] (Supplementary Figure S1).

### 3.4 PLR for predicting poor outcome and mortality

**Figure 3:**
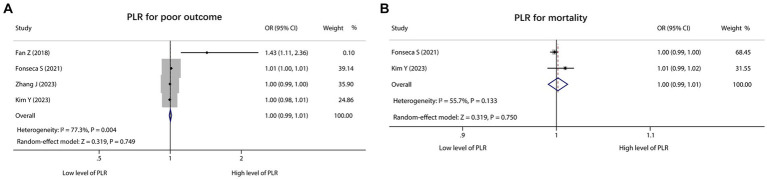
Forest plot of PLR for predicting poor outcome and mortality in ICH patients. Association of PLR with poor outcome **(A)** and mortality **(B)** in ICH patients.

There were 4 studies that reported PLR for predicting poor outcome. Heterogeneity existed among these studies (*I*^2^ = 77.3%, *p* = 0.004). According to the pooled analysis, PLR was not associated with poor outcome in ICH patients [OR (95% CI): 1.00 (0.99, 1.01), *p* = 0.749] (Figure 3A). In addition, 2 studies reported PLR for predicting mortality, and there was no heterogeneity existed among these studies (*I*^2^ = 55.7%, *p* = 0.133). Notably, the pooled analysis showed that PLR was also not correlated with mortality in ICH patients [OR (95% CI): 1.00 (0.99, 1.01), *p* = 0.750] (Figure 3B).

### 3.5 WBC for predicting poor outcome and mortality

**Figure 4:**
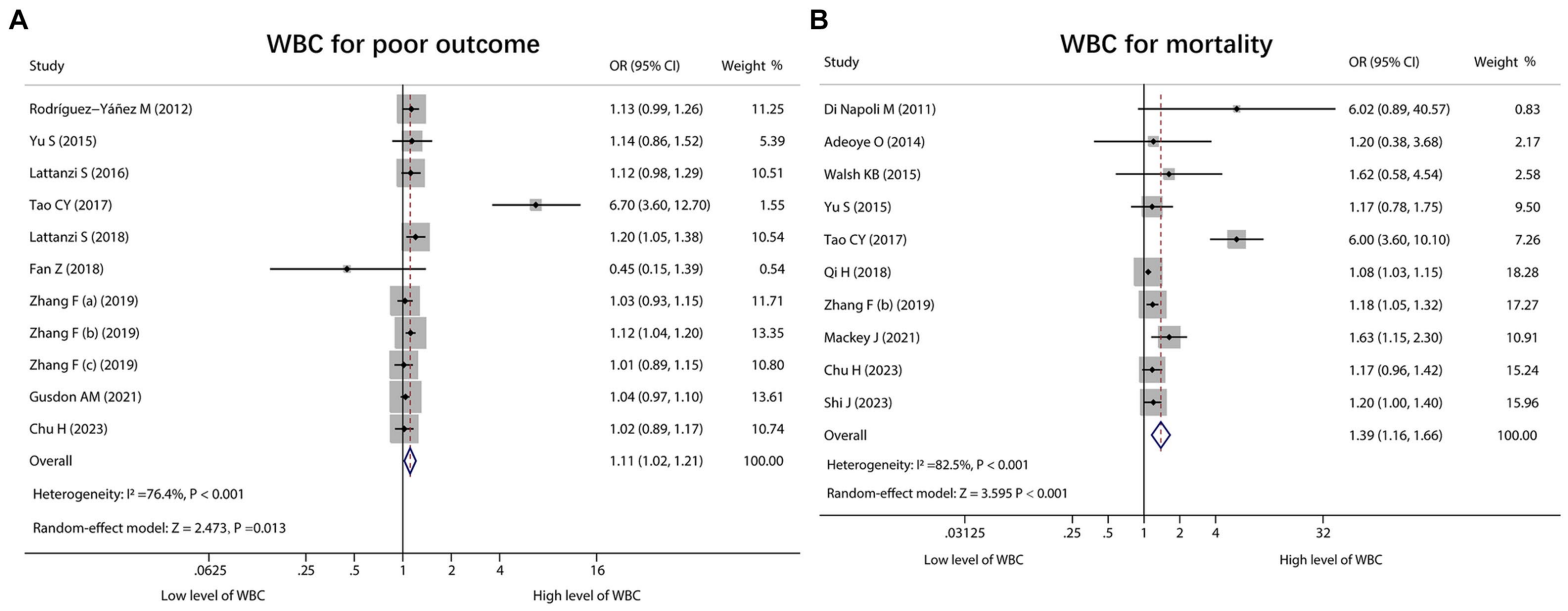
Forest plot of WBC for predicting poor outcome and mortality in ICH patients. Relationship of WBC with poor outcome **(A)** and mortality **(B)** in ICH patients.

WBC for estimating poor outcome was reported in 11 studies, and heterogeneity existed among these studies (*I*^2^ = 76.4%, *p* < 0.001). The pooled analysis exhibited that the high level of WBC was linked with poor outcome in ICH patients [OR (95% CI): 1.11 (1.02, 1.21), *p* = 0.013] (Figure 4A). Regarding WBC for predicting mortality, 10 studies reported that. Heterogeneity existed among these studies (*I*^2^ = 82.5%, *p* < 0.001). After conducting the pooled analysis, it was discovered that the high level of WBC was linked to increased mortality in ICH patients [OR (95% CI): 1.39 (1.16, 1.66), *p* < 0.001] (Figure 4B).

### 3.6 CRP for predicting poor outcome and mortality

**Figure 5:**
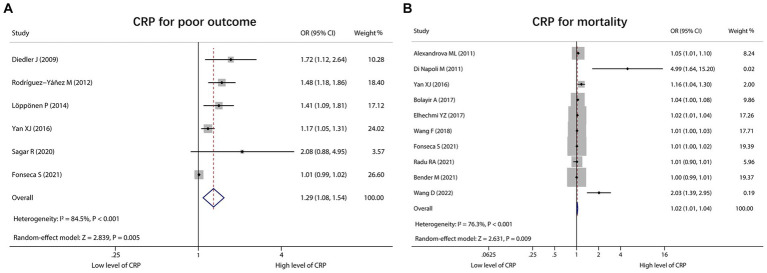
Forest plot of CRP for predicting poor outcome and mortality in ICH patients. Relationship of CRP with poor outcome **(A)** and mortality **(B)** in ICH patients.

A total of 6 studies reported CRP for predicting poor outcome, and heterogeneity existed among these studies (*I*^2^ = 84.5%, *p* < 0.001). The pooled analysis indicated that the high level of CRP was correlated with poor outcome in ICH patients [OR (95% CI): 1.29 (1.08, 1.54), *p* = 0.005] (Figure 5A). Moreover, 10 studies reported CRP for forecasting mortality. Heterogeneity existed among these studies (*I*^2^ = 76.3%, *p* < 0.001). The pooled analysis disclosed that CRP was associated with raised mortality in ICH patients [OR (95% CI): 1.02 (1.01, 1.04), *p* = 0.009] (Figure 5B).

### 3.7 Sensitivity analysis and quality assessment

Sensitivity analysis disclosed that omitting Fonseca would affect the result of PLR for estimating mortality. Apart from that, omitting any of a single study would not influence the results of the pooled analysis, which indicated the stability of this meta-analysis (Supplementary Table S1).

The included studies were evaluated by the Newcastle-Ottawa Scale criteria, and the total score of bias risk of each study ranged from 7–9, which indicated the low risk of bias in the included studies (Table 2).

**Table 2:** Quality assessment by Newcastle-Ottawa Scale criteria.

Included studies	Selection	Comparability	Outcome	Total score
Diedler et al. (31)	3	2	3	8
Alexandrova and Danovska (32)	4	2	2	8
Di Napoli et al. (33)	4	1	2	7
Rodríguez-Yáñez et al. (34)	3	2	2	7
Löppönen et al. (35)	4	1	2	7
Adeoye et al. (36)	4	2	3	9
Walsh et al. (37)	3	2	3	8
Yu et al. (38)	3	2	3	8
Lattanzi et al. (39)	3	1	3	7
Wang et al. (40)	4	1	2	7
Yan et al. (24)	3	2	2	7
Giede Jeppe et al. (41)	3	2	2	7
Tao et al. (25)	3	2	2	7
Sun et al. (42)	4	2	2	8
Bolayir et al. (43)	4	1	3	8
Elhechmi et al. (44)	4	2	3	9
Lattanzi et al. (45)	4	1	2	7
Fan et al. (46)	3	1	3	7
Wang et al. (47)	3	2	2	7
Qi et al. (48)	4	2	2	8
Zhang et al. (49)	3	2	3	8
Guo et al. (50)	4	1	3	8
Qin et al. (51)	3	2	3	8
Wang et al. (52)	3	2	3	8
Zhang et al. (53)	3	1	3	7
Zhang et al. (54)	4	2	2	8
Zhang et al. (55)	4	2	3	9
Chen et al. (56)	4	2	3	9
Sagar et al. (57)	3	2	2	7
Menon et al. (58)	3	1	3	7
Gusdon et al. (59)	4	1	2	7
Fonseca et al. (26)	4	2	3	9
Mackey et al. (60)	3	1	3	7
Li et al. (61)	4	2	2	8
Radu et al. (62)	4	2	2	8
Yang et al. (63)	4	2	2	8
Bender et al. (64)	3	2	2	7
Luo et al. (65)	3	2	2	7
Zhao et al. (27)	4	2	2	8
Du et al. (66)	4	2	3	9
Wang et al. (67)	4	2	2	8
Chu et al. (68)	3	2	3	8
Zhang et al. (69)	4	1	2	7
Zhang et al. (70)	3	2	2	7
Shi et al. (71)	3	2	3	8
Kim et al. (72)	3	1	3	7

### 3.8 Subgroup analysis for poor outcome based on study type and follow-up duration

The pooled analysis suggested that the high level of NLR was related to poor outcome in retrospective studies [OR (95% CI): 1.22 (1.14, 1.31), *p* < 0.001], studies with a follow-up duration of <90 days [OR (95% CI): 1.23 (1.08, 1.40), *p* = 0.002], and studies with a follow-up duration of ≥90 days [OR (95% CI): 1.20 (1.11, 1.29), *p* < 0.001]. Heterogeneity existed among these studies that reported NLR for predicting poor outcome (all *I*^2^ > 50.0%, *p* < 0.001) (Table 3).

**Table 3:** Subgroup analysis of the association of inflammation indexes with poor outcome.

Subgroup	Number of studies	*I* ^2^	*p*-value of heterogeneity	Effect model	OR (95% CI)	Z	*P*-value of statistic
NLR							
Total	22	84.1%	<0.001	Random	1.20 (1.13–1.27)	5.850	<0.001
Design							
Retrospective	18	85.8%	<0.001	Random	1.22 (1.14–1.31)	5.819	<0.001
Prospective	4	72.7%	0.012	Random	1.10 (0.93–1.29)	1.118	0.264
Follow-up							
<90 days	6	88.2%	<0.001	Random	1.23 (1.08–1.40)	3.040	0.002
≥90 days	16	83.0%	<0.001	Random	1.20 (1.11–1.29)	4.823	<0.001
PLR							
Total	4	77.3%	<0.001	Random	1.00 (0.99–1.01)	0.319	0.749
Design							
Retrospective	3	82.3%	0.001	Random	1.00 (1.00–1.02)	0.576	0.565
Prospective	1	(−)	(−)	(−)	(−)	(−)	(−)
Follow-up							
<90 days	1	(−)	(−)	(−)	(−)	(−)	(−)
≥90 days	3	70.2%	<0.001	Random	1.00 (0.99–1.02)	0.460	0.646
WBC							
Total	11	76.4%	<0.001	Random	1.11 (1.02–1.21)	2.473	0.013
Design							
Retrospective	10	77.6%	<0.001	Random	1.13 (1.02–1.25)	2.258	0.018
Prospective	1	(−)	(−)	(−)	(−)	(−)	(−)
Follow-up							
<90 days	3	0.0%	0.418	Fixed	1.06 (0.99–1.13)	1.599	0.110
≥90 days	8	82.5%	<0.001	Random	1.15 (1.02–1.30)	2.300	0.021
CRP							
Total	6	84.5	<0.001	Random	1.29 (1.08–1.54)	2.839	0.005
Design							
Retrospective	3	88.0%	<0.001	Random	1.32 (0.93–1.86)	1.553	0.120
Prospective	3	36.8%	0.205	Fixed	1.22 (1.10–1.35)	3.801	<0.001
Follow-up							
<90 days	1	(−)	(−)	(−)	(−)	(−)	(−)
≥90 days	5	81.6%	<0.001	Random	1.23 (1.03–1.47)	2.338	0.019

OR, odds ratio; CI, confidence interval; NLR, neutrophil-to-lymphocyte ratio; PLR, platelet-to-lymphocyte ratio; WBC, white blood cell count; CRP, C-reactive protein.

No correlation was found between PLR and poor outcome in retrospective studies and studies with a follow-up duration of ≥90 days (both *p* > 0.05) (Table 3).

The pooled analysis disclosed that the high level of WBC was correlated with poor outcome in retrospective studies [OR (95% CI): 1.13 (1.02, 1.25), *p* = 0.018] and studies with a follow-up duration of ≥90 days [OR (95% CI): 1.15 (1.02, 1.30), *p* = 0.021]. Heterogeneity existed among these studies that reported WBC for estimating poor outcome (both *I*^2^ > 50.0%, *p* < 0.001) (Table 3).

The pooled analysis displayed that the high level of CRP was associated with poor outcome in prospective studies [OR (95% CI): 1.22 (1.10, 1.35), *p* < 0.001] without heterogeneity among these studies (*I*^2^ = 36.8%, *p* = 0.205). In studies with a follow-up duration of ≥90 days, the high level of CRP was associated with poor outcome [OR (95% CI): 1.23 (1.03, 1.47), *p* = 0.019] with heterogeneity among these studies (*I*^2^ = 81.6%, *p* < 0.001) (Table 3).

### 3.9 Subgroup analysis for mortality based on study type and follow-up duration

The pooled analysis revealed that the high level of NLR was related to increased mortality in retrospective studies [OR (95% CI): 1.05 (1.02, 1.09), *p* = 0.007], prospective studies [OR (95% CI): 1.16 (1.08, 1.24), *p* < 0.001], studies with a follow-up duration of <90 days [OR (95% CI): 1.05 (1.01, 1.10), *p* = 0.021], and studies with a follow-up duration of≥90 days [OR (95% CI): 1.15 (1.03, 1.28), *p* = 0.012]. In terms of NLR for predicting mortality, heterogeneity existed among retrospective studies, studies with a follow-up duration of <90 days, and studies with a follow-up duration of≥90 days (all *I*^2^ > 50.0%, *p* < 0.001); heterogeneity did not exist in prospective studies (*I*^2^ = 0.0%, *p* = 0.651) (Table 4).

**Table 4:** Subgroup analysis of the association of inflammation indexes with mortality.

Subgroup	Number of studies	*I* ^2^	*P*-value of heterogeneity	Effect model	OR (95% CI)	Z	*P*-value of statistic
NLR							
Total	18	80.0%	<0.001	Random	1.06 (1.02–1.10)	3.186	<0.001
Design							
Retrospective	16	79.9%	<0.001	Random	1.05 (1.02–1.09)	2.716	0.007
Prospective	2	0.0%	0.651	Fixed	1.16 (1.08–1.24)	4.187	<0.001
Follow-up							
<90 days	12	77.2%	<0.001	Random	1.05 (1.01–1.10)	2.307	0.021
≥90 days	6	84.7%	<0.001	Random	1.15 (1.03–1.28)	2.512	0.012
PLR							
Total	2	55.7%	0.133	Random	1.00 (0.99–1.01)	0.319	0.750
Design							
Retrospective	1	(−)	(−)	(−)	(−)	(−)	(−)
Prospective	1	(−)	(−)	(−)	(−)	(−)	(−)
Follow-up							
<90 days	2	55.7%	0.133	Random	1.00 (0.99–1.01)	0.319	0.750
≥90 days	0	(−)	(−)	(−)	(−)	(−)	(−)
WBC							
Total	10	82.5%	<0.001	Random	1.39 (1.16–1.66)		
Design							
Retrospective	8	85.4%	<0.001	Random	1.36 (1.14–1.63)	3.370	0.001
Prospective	2	28.9%	0.236	Fixed	2.18 (0.88–5.38)	1.683	0.092
Follow-up							
<90 days	6	13.1%	0.331	Fixed	1.25 (1.11–1.40)	3.648	<0.001
≥90 days	4	93.0%	<0.001	Random	1.51 (1.11–2.04)	2.639	0.008
CRP							
Total	10	76.3%	<0.001	Random	1.02 (1.01–1.04)	2.631	0.009
Design							
Retrospective	7	71.4%	0.002	Random	1.02 (1.00–1.03)	2.145	0.032
Prospective	3	80.1%	0.007	Random	1.14 (0.96–1.37)	1.477	0.140
Follow-up							
<90 days	9	74.9%	<0.001	Random	1.02 (1.00–1.04)	2.385	0.017
≥90 days	1	(−)	(−)	(−)	(−)	(−)	(−)

OR, odds ratio; CI, confidence interval; NLR, neutrophil-to-lymphocyte ratio; PLR, platelet-to-lymphocyte ratio; WBC, white blood cell count; CRP, C-reactive protein.

No correlation was found between PLR and mortality in studies with follow-up duration of <90 days (*p* > 0.05) (Table 4).

The pooled analysis disclosed that the high level of WBC was correlated with raised mortality in retrospective studies [OR (95% CI): 1.36 (1.14, 1.63), *p* = 0.001], studies with a follow-up duration of <90 days [OR (95% CI): 1.25 (1.11, 1.40), *p* < 0.001], and studies with a follow-up duration of ≥90 days [OR (95% CI): 1.51 (1.11, 2.04), *p* = 0.008]. Regarding WBC for forecasting mortality, heterogeneity existed among retrospective studies and studies with a follow-up duration of ≥90 days (both *I*^2^ > 50.0%, *p* < 0.001). But it did not exist in studies with a follow-up duration of <90 days (*I*^2^ = 13.1%, *p* = 0.331) (Table 4).

The pooled analysis showed that the high level of CRP was associated with elevated mortality in retrospective studies [OR (95% CI): 1.02 (1.00, 1.03), *p* = 0.032] and studies with a follow-up duration of <90 days [OR (95% CI): 1.02 (1.00, 1.04), *p* = 0.017]. Heterogeneity existed among these studies that reported CRP for estimating mortality (both *I*^2^ > 50.0%, *p* < 0.01) (Table 4).

### 3.10 Subgroup analysis for the association between NLR and poor outcome based on sampling time

In studies with a sampling time at admission, 20 studies reported NLR for predicting poor outcome, and heterogeneity existed among these studies (*I*^2^ = 84.4%, *p* < 0.001). The random effect model exhibited that the high level of NLR was correlated with poor outcome [OR (95% CI): 1.19 (1.12, 1.27), *p* < 0.001]. In studies with a sampling time within 48 h after surgery, 2 studies reported NLR for predicting poor outcome, and there was no heterogeneity in these studies (*I*^2^ = 58.2%, *p* = 0.122). The random effect model suggested that the high level of NLR was related to a poor outcome [OR (95% CI): 1.24 (1.06, 1.44), *p* = 0.007] (Supplementary Table S2).

### 3.11 Publication bias

**Figure 6:**
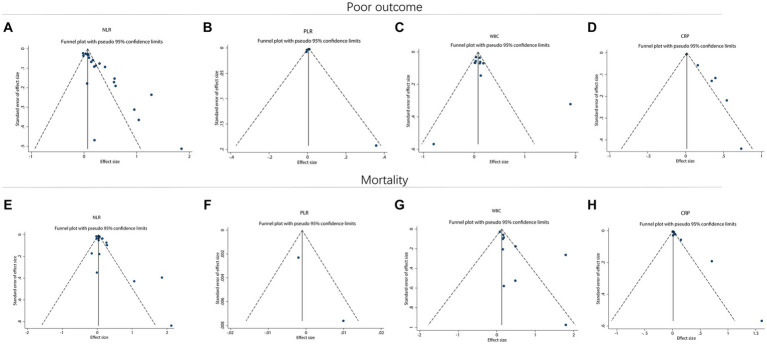
Funnel plot for publication bias. Funnel plot of NLR **(A)**, PLR **(B)**, WBC **(C)**, and CRP **(D)** for predicting poor outcome in ICH patients. Funnel plot of NLR **(E)**, PLR **(F)**, WBC **(G)**, and CRP **(H)** for predicting mortality in ICH patients.

Funnel plots suggested that there might be a potential publication bias in NLR for predicting poor outcome (Figure 6A). However, publication bias might not exist in PLR (Figure 6B) and WBC (Figure 6C) for estimating poor outcome. Notably, potential publication bias might also exist in CRP for forecasting poor outcome (Figure 6D). In terms of mortality, NLR (Figure 6E), PLR (Figure 6F), and WBC (Figure 6G) for predicting mortality might have a low risk of publication bias. However, CRP for estimating mortality might have a high risk of publication bias (Figure 6H). Begg’s test disclosed that only NLR for predicting poor outcome (*p* < 0.001) and CRP for predicting mortality (*p* = 0.007) existed publication bias. Subsequently, the trim-and-filling method was applied to validate the stability, and it was found that the OR (95% CI) of NLR for estimating poor outcome before and after filling imputed missing studies was 0.07 (0.05–0.09) (*p* < 0.001) and 1.05 (1.03–1.07) (*p* < 0.001), which indicated the model was robust. Meanwhile, the OR (95% CI) of CRP for estimating mortality before and after filling imputed missing studies was 0.01 (0.01–0.02) (*p* < 0.001) and 1.01 (1.01–1.02) (*p* < 0.001), which indicated the model was stable.

## 4 Discussion

Aggravated inflammation facilitates the progression of secondary brain injury, which may ultimately contribute to poor outcome in ICH patients (16). In this meta-analysis, it was discovered that the high level of NLR, WBC, and CRP were related to poor outcome in ICH patients. The potential reasons might be that: (1) after ICH, the neutrophils would rapidly reach the hemorrhage site and infiltrate the brain parenchyma, which impaired the blood–brain barrier and led to neurological injury, thereby resulting in poor outcome (16). In addition, the inflammatory response following ICH would further interfere with the function of the innate and adaptive immune cells, which might lead to adaptive immunosuppression (73–75). (2) increased leukocytes could also facilitate the neurotoxicity through production of matrix metalloproteinases, reactive oxygen species, and tumor necrosis factor (TNF)-α, which further contributed brain injury (19). (3) CRP could activate the complement cascade and microglia, and promote the release of proinflammatory cytokines to aggravate secondary brain injury, which ultimately contributed to poor outcome (17, 76, 77). Taken together, considering the involvement of neutrophils, lymphocytes, and CRP in the brain injury after ICH, NLR, WBC, and CRP might have the ability to predict the poor outcome. Notably, heterogeneity existed among the studies that reported the correlation of NLR, WBC, and CRP with poor outcome in ICH patients. Therefore, the findings of this meta-analysis needed further validation.

Some studies also disclose the role of NLR, PLR, WBC, and CRP in forecasting mortality in ICH patients (25, 26, 33, 47). For instance, the high level of NLR independently predicts higher mortality in ICH patients (47). Meanwhile, the high level of WBC is also independently linked with increased mortality in ICH patients (25). Furthermore, another study indicates that the high level of CRP can estimate elevated mortality in ICH patients (33). However, one study figures out that PLR lacks the ability to predict mortality in ICH patients (26). In this meta-analysis, it was found that the high level of NLR, WBC, and CRP were correlated with increased mortality in ICH patients. The possible reasons might be that: (1) following ICH, neutrophils would impair the blood–brain barrier and induce neurological injury, which might further induce temporary immune suppression and lead to lymphocytopenia (16, 74, 78). Subsequently, lymphocytopenia would increase the risk of infection, which was responsible for mortality (73, 79). Therefore, the high level of NLR predicted elevated mortality in ICH patients. (2) the high level of NLR, WBC, and CRP could reflect exacerbated inflammatory status, and aggravated inflammation could facilitate hematoma expansion after ICH (80, 81). Then the expanded hematoma would further lead to intracranial hypertension, resulting in mortality (81). Conclusively, the high level of NLR, WBC, and CRP predicted raised mortality in ICH patients.

Further subgroup analysis discovered that in retrospective studies, the high level of NLP and WBC were related to poor outcome; meanwhile, the high level of NLR, WBC, and CRP were correlated with increased mortality in ICH patients. However, in prospective studies, only the high level of CRP estimated poor outcome, and only the high level of NLR predicted elevated mortality in ICH patients. A possible reason would be that selection bias and information bias would exist in retrospective studies, which might influence the prognostic effect of these inflammatory indexes (82, 83). Therefore, the findings of this meta-analysis should be read with caution, and more solid evidence was required. Apart from study design, subgroup analysis based on follow-up duration disclosed that in studies with a follow-up duration of ≥90 days, the high level of NLR, WBC, and CRP was related to poor outcome; the high level of NLR and WBC was correlated with increased mortality in ICH patients. In studies with a follow-up duration of <90 days, only the high level of NLR was linked to poor outcome, and the high level of NLR, WBC, and CRP was linked with raised mortality in ICH patients. A potential reason might be that aggravated inflammation after ICH might sustainably degrade immune resilience over time, which increased the risk of infection and obstructed the recovery from the disease, contributing to a poor outcome and increased mortality (84, 85). Considering that the longer follow-up duration might more objectively reflect the prognosis of ICH patients, it was speculated that the high level of NLR, WBC, and CRP had a good ability to predict poor outcome, and the high level of NLR and WBC could estimate increased mortality in ICH patients. However, more evidence was required to validate this speculation. Notably, limited by the number of studies, whether the prognostic effect of PLR and CRP would be affected by follow-up duration should be further studied. In addition, other factors, such as hematoma size, surgery, co-infections, etc., might also affect the prognosis of ICH patients, which could be a study direction for subsequent studies. Moreover, this meta-analysis also discovered that in studies with a sampling time at admission and within 48 h after surgery, the high level of NLR was correlated with poor outcome. Based on this finding, it was speculated that the ability of NLR to predict poor outcome in ICH patients was not affected by the sampling times. However, limited by the sample size of this meta-analysis, the number of studies that could be included in the subgroup analysis was small, especially for the studies in which the sampling times were not at admission. Therefore, the findings of this meta-analysis should be further validated.

Limitations could not be omitted in this meta-analysis. Firstly, the regions of the included studies differed, and most included studies were conducted in China. Thus, the generalization of the findings of this meta-analysis should be validated. Secondly, some studies had different sampling times, which might affect the results. Thirdly, many screened studies had a retrospective design; thus, selection bias and information bias might exist. Fourthly, some factors, such as sampling time and follow-up duration, would affect the role of PLR in predicting the prognosis of ICH patients. In addition, the number of studies that reported PLR for predicting poor outcome (*N* = 4) and mortality (*N* = 2) in ICH patients was relatively small, which limited the statistical power and the conduction of relevant subgroup analyses. Therefore, more evidence was required to validate the prognostic implication of PLR in ICH patients. Fifthly, aneurysmal cerebral hemorrhage should be excluded due to differences in etiology. However, only Radu and Kim clearly indicated that they excluded aneurysmal cerebral hemorrhage patients, while other studies did not provide this information. Therefore, the findings of this meta-analysis should be further validated.

This meta-analysis concludes that the high level of NLR, WBC, and CRP estimates poor outcome and elevated mortality in ICH patients. Although these indexes are dynamically changing, in our opinion, their variation is still within an abnormal range. Therefore, the high level of NLR, WBC, and CRP could still indicate aggravated inflammation after ICH. Clinically, given that the detection of NLR, WBC, and CRP is simple and the high level of these indexes may provide prognostic information of ICH patients, the detection of these indexes should be widely applied in ICH patients. In addition, considering the high level of NLR, WBC, and CRP could reflect aggravated inflammation, acute interventions that target inflammation may help to improve the prognosis of ICH patients.

## Data availability statement

The original contributions presented in the study are included in the article/Supplementary material, further inquiries can be directed to the corresponding author.

## Author contributions

PG: Conceptualization, Data curation, Formal analysis, Investigation, Methodology, Writing – original draft, Writing – review & editing. WZ: Conceptualization, Data curation, Formal analysis, Project administration, Supervision, Validation, Writing – original draft, Writing – review & editing.
