# Supplementary material for: Neutrophil-to-lymphocyte ratio, white blood cell, and C-reactive protein predicts poor outcome and increased mortality in intracerebral hemorrhage patients: a meta-analysis
Source: Front Neurol. 2024 Jan 15;14:1288377. doi: 10.3389/fneur.2023.1288377 (PMC10824245; doi:10.3389/fneur.2023.1288377)
Supplement: Supplementary file 1 [file Table_1.docx]

**Supplementary Table 1.** Sensitivity analysis.

| Study omitted | Estimate (OR) | 95% CI | |
| --- | --- | --- | --- |
|  |  | Lower | Upper |
| **Poor outcomes** |  |  |  |
| NLR |  |  |  |
| Lattanzi S (2016) | 1.19 | 1.12 | 1.26 |
| Giede Jeppe A (2017) | 1.22 | 1.14 | 1.30 |
| Tao CY (2017) | 1.17 | 1.10 | 1.23 |
| Sun Y (2017) | 1.19 | 1.12 | 1.26 |
| Lattanzi S (2018) | 1.18 | 1.11 | 1.25 |
| Fan Z (2018) | 1.18 | 1.12 | 1.26 |
| Zhang F (2018) | 1.20 | 1.13 | 1.27 |
| Guo R (2019) | 1.20 | 1.13 | 1.27 |
| Qin J (2019) | 1.22 | 1.14 | 1.30 |
| Zhang F (a) (2019) | 1.21 | 1.14 | 1.30 |
| Zhang F (b) (2019) | 1.20 | 1.13 | 1.28 |
| Zhang F (c) (2019) | 1.18 | 1.11 | 1.25 |
| Menon G (2021) | 1.18 | 1.12 | 1.26 |
| Gusdon AM (2021) | 1.22 | 1.14 | 1.30 |
| Fonseca S (2021) | 1.20 | 1.12 | 1.27 |
| Li J (2021) | 1.22 | 1.14 | 1.30 |
| Yang W (2021) | 1.22 | 1.14 | 1.31 |
| Luo S (2022) | 1.22 | 1.14 | 1.30 |
| Zhao Y (2022) | 1.19 | 1.12 | 1.26 |
| Du Y (2022) | 1.19 | 1.12 | 1.26 |
| Zhang J (2023) | 1.20 | 1.13 | 1.28 |
| Kim Y (2023) | 1.21 | 1.13 | 1.29 |
| Combined | 1.20 | 1.13 | 1.27 |
| PLR |  |  |  |
| Fan Z (2018) | 1.00 | 0.99 | 1.01 |
| Fonseca S (2021) | 1.00 | 0.98 | 1.01 |
| Zhang J (2023) | 1.00 | 0.99 | 1.02 |
| Kim Y (2023) | 1.00 | 0.99 | 1.02 |
| Combined | 1.00 | 0.99 | 1.01 |
| WBC |  |  |  |
| Rodríguez-Yáñez M (2012) | 1.11 | 1.01 | 1.22 |
| Yu S (2015) | 1.11 | 1.02 | 1.21 |
| Lattanzi S (2016) | 1.11 | 1.01 | 1.22 |
| Tao CY (2017) | 1.08 | 1.04 | 1.12 |
| Lattanzi S (2018) | 1.10 | 1.01 | 1.20 |
| Fan Z (2018) | 1.11 | 1.03 | 1.21 |
| Zhang F (a) (2019) | 1.12 | 1.02 | 1.23 |
| Zhang F (b) (2019) | 1.12 | 1.01 | 1.23 |
| Zhang F (c) (2019) | 1.12 | 1.03 | 1.23 |
| Gusdon AM (2021) | 1.13 | 1.02 | 1.25 |
| Chu H (2023) | 1.12 | 1.03 | 1.23 |
| Combined | 1.11 | 1.02 | 1.21 |
| CRP |  |  |  |
| Diedler J (2009) | 1.24 | 1.04 | 1.48 |
| Rodríguez-Yáñez M (2012) | 1.23 | 1.03 | 1.47 |
| Löppönen P (2014) | 1.26 | 1.05 | 1.52 |
| Yan XJ (2016) | 1.38 | 1.05 | 1.82 |
| Sagar R (2020) | 1.26 | 1.06 | 1.51 |
| Fonseca S (2021) | 1.37 | 1.17 | 1.62 |
| Combined | 1.29 | 1.08 | 1.54 |
| **Mortality** |  |  |  |
| NLR |  |  |  |
| Wang F (2016) | 1.06 | 1.03 | 1.11 |
| Giede Jeppe A (2017) | 1.07 | 1.03 | 1.11 |
| Tao CY (2017) | 1.05 | 1.02 | 1.09 |
| Sun Y (2017) | 1.06 | 1.02 | 1.10 |
| Wang F (2018) | 1.06 | 1.02 | 1.10 |
| Qi H (2018) | 1.07 | 1.02 | 1.11 |
| Wang F (2019) | 1.07 | 1.02 | 1.11 |
| Zhang F (b) (2019) | 1.05 | 1.02 | 1.09 |
| Chen W (2020) | 1.07 | 1.02 | 1.11 |
| Fonseca S (2021) | 1.07 | 1.03 | 1.11 |
| Mackey J (2021) | 1.06 | 1.02 | 1.10 |
| Li J (2021) | 1.07 | 1.03 | 1.12 |
| Radu RA (2021) | 1.06 | 1.02 | 1.11 |
| Yang W (2021) | 1.06 | 1.02 | 1.11 |
| Du Y (2022) | 1.06 | 1.02 | 1.10 |
| Zhang G (2023) | 1.07 | 1.02 | 1.11 |
| Shi J (2023) | 1.05 | 1.01 | 1.09 |
| Kim Y (2023) | 1.05 | 1.01 | 1.09 |
| Combined | 1.06 | 1.02 | 1.10 |
| PLR |  |  |  |
| Fonseca S (2021) | 1.01 | 1.00 | 1.03 |
| Kim Y (2023) | 1.00 | 0.99 | 1.00 |
| Combined | 1.00 | 0.99 | 1.01 |
| WBC |  |  |  |
| Di Napoli M (2011) | 1.37 | 1.15 | 1.63 |
| Adeoye O (2014) | 1.39 | 1.16 | 1.67 |
| Walsh KB (2015) | 1.38 | 1.15 | 1.66 |
| Yu S (2015) | 1.42 | 1.17 | 1.72 |
| Tao CY (2017) | 1.17 | 1.08 | 1.26 |
| Qi H (2018) | 1.52 | 1.19 | 1.95 |
| Zhang F (b) (2019) | 1.49 | 1.17 | 1.90 |
| Mackey J (2021) | 1.36 | 1.13 | 1.63 |
| Chu H (2023) | 1.45 | 1.18 | 1.78 |
| Shi J (2023) | 1.45 | 1.17 | 1.79 |
| Combined | 1.39 | 1.16 | 1.66 |
| CRP |  |  |  |
| Alexandrova ML (2011) | 1.02 | 1.00 | 1.04 |
| Di Napoli M (2011) | 1.02 | 1.01 | 1.04 |
| Yan XJ (2016) | 1.02 | 1.00 | 1.04 |
| Bolayir A (2017) | 1.02 | 1.00 | 1.04 |
| Elhechmi YZ (2017) | 1.02 | 1.00 | 1.04 |
| Wang F (2018) | 1.03 | 1.01 | 1.05 |
| Fonseca S (2021) | 1.03 | 1.01 | 1.05 |
| Radu RA (2021) | 1.02 | 1.01 | 1.04 |
| Bender M (2021) | 1.03 | 1.01 | 1.05 |
| Wang D (2022) | 1.02 | 1.01 | 1.03 |
| Shi J (2023) | 1.02 | 1.01 | 1.04 |
| Combined | 1.02 | 1.01 | 1.04 |

OR, odds ratio; CI, confidence interval; NLR, neutrophil-to-lymphocyte ratio; PLR, platelet-to-lymphocyte ratio; WBC, white blood cell count; CRP, C-reactive protein.
