# Supplementary material for: Neutrophil-to-lymphocyte ratio, white blood cell, and C-reactive protein predicts poor outcome and increased mortality in intracerebral hemorrhage patients: a meta-analysis
Source: Front Neurol. 2024 Jan 15;14:1288377. doi: 10.3389/fneur.2023.1288377 (PMC10824245; doi:10.3389/fneur.2023.1288377)
Supplement: Supplementary file 2 [file Table_2.docx]

**Supplementary Table 2.** Subgroup analysis of the association of NLR sampling time with poor outcome.

| Subgroup | Number of studies | I^2^ | *P*-value of heterogeneity | Effect model | OR (95% CI) | Z | *P*-value of statistic |
| --- | --- | --- | --- | --- | --- | --- | --- |
| Total NLR | 22 | 84.1% | <0.001 | Random | 1.20 (1.13-1.27) | 5.850 | <0.001 |
| **Sampling time** |  |  |  |  |  |  |  |
| Admission | 20 | 84.4% | <0.001 | Random | 1.19 (1.12-1.27) | 5.407 | <0.001 |
| Within 48h after surgery | 2 | 58.2% | 0.122 | Random | 1.24 (1.06-1.44) | 2.699 | 0.007 |

NLR, neutrophil-to-lymphocyte ratio; OR, odds ratio; CI, confidence interval.
