# Supplementary figures and images for: Neutrophil-to-lymphocyte ratio, white blood cell, and C-reactive protein predicts poor outcome and increased mortality in intracerebral hemorrhage patients: a meta-analysis
Source: Front Neurol. 2024 Jan 15;14:1288377. doi: 10.3389/fneur.2023.1288377 (PMC10824245; doi:10.3389/fneur.2023.1288377)

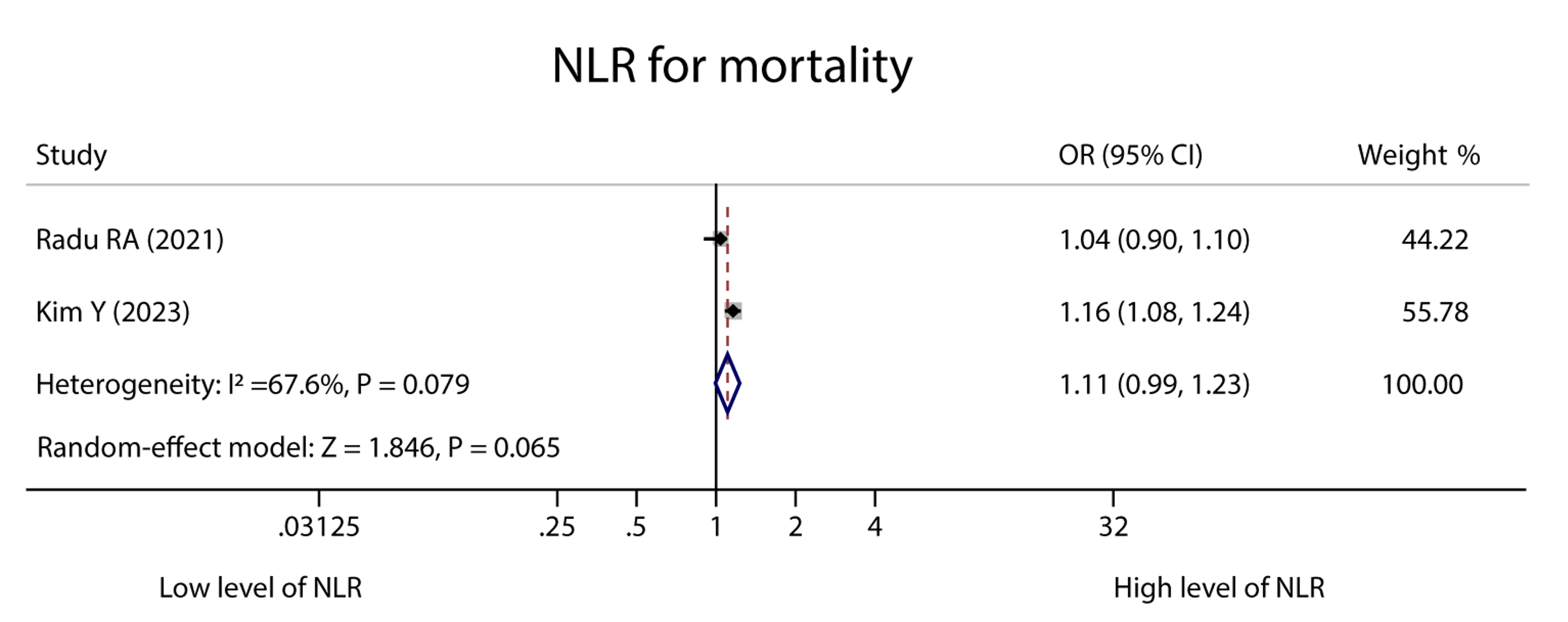

Supplement: Supplementary file 3 [file Image_1.TIF]
